# Conferences at the Nursing and Midwifery Exhibition

**Published:** 1921-05-28

**Authors:** 


### CONFERENCES AT THE NURSING AND MIDWIFERY EXHIBITION.

The afternoons and evenings of Wednesday, Thursday, and Friday, May 18 to 20, were devoted to conferences on a variety of subjects, at some of which papers were read and at all of which discussion was invited. A large meeting assembled to hear Major Simonds, M.A., Secretary of the Poor-law Officers' Association, on "Superannuation under the Poor-law." Despite certain objections in the Poor-law Officers' Superannuation Act of 1896, he claimed that it compares very favourably with the superannuation schemes in other services. According to the forty-third annual report of the Local Government Board, the contributions of officers during the year 1914 under this Act amounted to £61,869, while the amount paid in superannuation allowances was £200,024, or a cost to the rates of £138,000 odd. It was to be assumed, he said, that the cost to the rates at the present time is far in excess of this sum.

In his address on "Pensions for Hospital Nurses," Mr. S. Harris, of the Clerical, Medical and General Life Assurance Society, dealt with actuarial considerations in regard to pensions, and pointed out difficulties in connection with individual superannuation schemes. Miss Ruth Young (Secretary of the Headmistresses' Association), speaking on "Nurses' Salaries from the Professional Women's Point of View," commanded sympathy for her plea for improvement both from the individual and the national standpoint, though some of her suggestions were held by the older members of the audience to be somewhat Utopian. At the following meeting Miss Halford (Secretary, National Association for the Prevention of Infant Mortality) touched on the subject of salaries in so far as the public-health worker is concerned; even to-day as low a salary as £120 is sometimes offered, though as a general rule in rural areas the salaries range from £150 to £200, while in London they begin at £200, with a few plums at £350. Dealing with the "Present and Future of Health Work," Miss Halford pointed out that this work possesses the advantages of fairly regular and not too long hours and living out, though it imposes considerable strain. The reason for the insufficient supply of health workers is that the right people do not have the right training; the health worker should have training in social service and routine work, and a three years' hospital training is not necessarily the best. There should be a special training for public-health nurses to enable them to deal with preventive rather than curative work. This point was strongly insisted on by Miss Halford throughout her clear address; the public-health nurse's training should be concerned with health rather than ill-health; she should be a health missioner (Florence Nightingale's term) rather than a nurse.

The meetings on Wednesday evening and Thursday afternoon were devoted to the Midwife, her Art and History. In the evening the Rev. A. Lombardini spoke on "The Value of Nurses' Leagues," of which that of the Kensington Infirmary is a good example, and Miss Grace Vaughan (Westminster District Nursing Association) discussed suggestions for a registered uniform. On Friday afternoon, with Miss L. A. Clark, Matron of Whipps Cross Hospital, in the chair, Lieut.-Col. N. Howard Mummery, M.R.C.S., General Secretary of the Federation of Medical and Allied Societies, under the title of "How the Nurse can make her Voice Heard," put forward, briefly and cogently, the claims of his Federation to act as a unifying organisation for its various constituents, whose independence and autonomy are absolutely preserved. With one exception, he said, every organisation in the nursing and midwifery professions is helping to form the Federation, and nearly every section of medical practice. In the past there has been no machinery in the medical or allied professions for influencing public opinion. There must be one voice, and that voice must be made up of the opinions definitely expressed by the various professions united to form it. The nurse who belongs to no organisation is selfish: she is not doing the best for herself and is doing nothing to further the interests of her profession. The Federation definitely stands behind the medical group in Parliament to help it to obtain information, and to keep it informed what the profession is thinking and wanting. He asked, if there are medical men in Parliament, why should not nurses and midwives be in Parliament also? The Medical Parliamentary Committee would welcome the co-operation of these professions in Parliament. Their duties, though not always parallel, are always ancillary to those of the medical man, and all these professions should be represented.

Miss Marsters (Superintendent of the Paddington District Nursing Association) explained the working in her district of the visiting nursing of the middle classes by district nurses, which she said has been successful and harmonious; it is not possible for the private visiting nurse to undertake such cases. Disappointment was caused by the non-appearance of Sisters Starkey and Monnet, from Southwark Hospital, who were down to speak on "The Eight-hour Day in Practice." Despite the Chairman's pleading, no one from the audience could be induced to give her views on the subject, but Miss Marsters gave her experience of an eight-hour day in district nursing, which should prove quite practicable. In the evening, under the chairmanship of Miss A. C. Gibson, late Matron of Birmingham Poor-law Infirmary, Sir Robert Armstrong Jones, C.B.E., dealt with "The Nursing of Nervous Patients," and Sir James Cantlie, K.B.E., that favourite of popular audiences, concluded with "Some Words to Nurses."

### Observation Tests.

The following are the prize-winners in the Observation Tests arranged by the conductors of the Exhibition:

*Two-Table Competition.*—Nurses A. E. M. Burstow, M. A. Oust, Phillips, R. Rogers, E. M. Jones, Joan Kutchley.

*One-Table Competition.*—Assistant Matron G. Wilkinson, Nurses Clarke, Oust, Violet Morphew, Jowett, Harris, and Sister Campbell.

It will be observed that Nurse Oust is a winner in both competitions.

